# Multiple Aspects of Gene Dysregulation in Huntington’s Disease

**DOI:** 10.3389/fneur.2013.00127

**Published:** 2013-10-23

**Authors:** Lara Moumné, Sandrine Betuing, Jocelyne Caboche

**Affiliations:** ^1^Laboratoire de Physiopathologie des Maladies du Système Nerveux Central, Neuronal Signaling and Gene Regulation, CNRS-UMR7224, INSERM-UMS952, Université Pierre et Marie Curie-Paris 6, Paris, France

**Keywords:** transcription, epigenetics, chromatin remodeling, histone modifications, REST, miRNAs

## Abstract

Huntington’s Disease (HD) is a genetic neurodegenerative disease caused by a CAG expansion in the gene encoding Huntingtin (*Htt*). It is characterized by chorea, cognitive, and psychiatric disorders. The most affected brain region is the striatum, and the clinical symptoms are directly correlated to the rate of striatal degeneration. The wild-type Htt is a ubiquitous protein and its deletion is lethal. Mutated (expanded) Htt produces excitotoxicity, mitochondrial dysfunctions, axonal transport deficit, altered proteasome activity, and gene dysregulation. Transcriptional dysregulation occurs at early neuropathological stages in HD patients. Multiple genes are dysregulated, with overlaps of altered transcripts between mouse models of HD and patient brains. Nuclear localization of Exp-Htt interferes with transcription factors, co-activators, and proteins of the transcriptional machinery. Another key mechanism described so far, is an alteration of cytoplasmic retention of the transcriptional repressor REST, which is normally associated with wild-type Htt. As such, Exp-Htt causes alteration of transcription of multiple genes involved in neuronal survival, plasticity, signaling, and mitochondrial biogenesis and respiration. Besides these transcriptional dysregulations, Exp-Htt affects the chromatin structure through altered post-translational modifications (PTM) of histones and methylation of DNA. Multiple alterations of histone PTM are described, including acetylation, methylation, ubiquitylation, polyamination, and phosphorylation. Exp-Htt also affects the expression and regulation of non-coding microRNAs (miRNAs). First multiple neural miRNAs are controlled by REST, and dysregulated in HD, with concomitant de-repression of downstream mRNA targets. Second, Exp-Htt protein or RNA may also play a major role in the processing of miRNAs and hence pathogenesis. These pleiotropic effects of Exp-Htt on gene expression may represent seminal deleterious effects in the pathogenesis of HD.

## Introduction

Huntington’s disease (HD) is a dominantly inherited genetic disorder induced by an abnormal expansion of a CAG trinucleotide repeat at the 5′ terminal part of the Huntingtin (Htt) gene leading to a polyglutamine expansion in the Htt protein (1). It is the most frequent genetic disease induced by a polyglutamine expansion with a prevalence of three to seven for 100,000 persons. Individuals with 39 CAG repeats or more will develop the clinical symptoms and signs of HD including neuropsychiatric, motor, and cognitive abnormalities that cause a progressive loss of functional capacity and shorten life span (2, 3). Intermediate alleles repetitions (between 36 and 39 repeats) are usually associated with late onset disease and may express a variable penetrance as the patient may die before disease onset (4–6). HD has a well-defined neuropathology, and informative pre-manifest predictive genetic testing. Brain weight may be reduced by as much as 25–30% in advanced HD cases. Gross pathology in HD is mainly observed in the brain, with atrophy predominating in the caudate-putamen, and to a lesser extent, the cerebral cortex. Furthermore, despite the early expression of mutated Htt (Exp-Htt) in all neuronal cells the first symptoms and neuropathological hallmarks appear at adulthood, around 40–45 years old. The age of onset of the disease is conversely proportional to the number of CAG repeats in the affected allele. Once the first symptoms have appeared, the disease progresses and leads progressively to death. As neuro-degeneration progresses in the striatum, the severity of symptoms increases (2). Magnetic resonance imaging (MRI) studies indicate that striatal atrophy begins up to 15 years before predicted onset and continues through the period of manifest illness (7). Therefore, the pre-symptomatic phase in HD provides a unique window for therapeutic intervention and neuro-protection.

The clinical features of HD can be divided into three groups: movement disorders, cognitive impairment, and psychiatric manifestations [see Ref. (8) for review]. Chorea is the most characteristic movement disorder of HD and is characterized by brief, involuntary, abnormal movements, which appear unpredictably in all the parts of the body. Cognitive impairment can precede motor symptoms or occur during the course of the disease, and usually leads, in turn, to dementia. Neurobehavioral symptoms include irritability, agitation, apathy, anxiety, social withdrawal, impulsiveness, alcohol abuse, obsessive-compulsive disorder. Mood disorders are very frequent, including depression and HD patients have a risk of suicide that is 10 times higher than in the general population.

There is no cure for HD, although medication can be given to help control the emotional and movement problems associated with HD. While medicines may help keep clinical symptoms under control, they are unable to stop or reverse the course of the disease.

Basic research has provided new insights into the complex cellular and molecular alterations involved in the pathogenesis of HD. The wild-type Htt is an ubiquitous protein, expressed in most cells and within all cellular compartments (9). It is required for normal embryonic development, and *Htt* knock-out mice show early lethality (E8,5) (10, 11). Furthermore, selective knock-down of the Htt protein in neurons and testis produces apoptosis in these tissues (12). Whether neuronal degeneration in HD is due to loss of normal function of wild-type Htt, or gain of toxic functions of Exp-Htt is still a debate. Expansion of polyglutamine in Htt leads to protein aggregation (9), a mechanism thought to be primarily involved in several neurological disorders caused by CAG repeats. It still remains to be established whether the mutant Htt aggregates are incidental, pathogenic, or neuroprotective. Expansion of polyglutamine in Htt produces by itself multiple cellular dysfunctions, including excitotoxicity, altered mitochondrial functions, axonal transport deficit, altered proteasome activity, and gene dysregulation, that were extensively described in other reviews (8, 13). Among these alterations, transcriptional dysregulation occurs at early neuropathological stages in HD and seems to be seminal in the neuropathological process.

## Transcriptional Dysregulation in HD

Dysregulation of transcription was first described in HD brain tissues at early neuropathological stages and then found in pre-symptomatic HD transgenic mice. Expression of enkephalin, substance P, dopamine D1 and D2 receptor mRNAs were shown to be altered in the caudate-putamen of HD patients in *post mortem* tissue in the early grade using *in situ* hybridization (14). Subsequently, cDNA microarray performed on genetically engineered HD mouse models allowed thousands of genes to be monitored, and provided a global genomic view of striatal dysfunctions in HD. From these analysis, neurotransmitter receptors, enzymes, and proteins involved in neuron structure, stress response, and axonal transport were found to be dysregulated (15–20). These changes were reproducibly observed in various HD mouse models and in the human HD caudate-putamen (19). Altogether these observations strongly supported that changes in transcription underlie neuro-degeneration rather than unspecific degradation of all RNAs in affected neurons.

Importantly, more than 81% of striatal-enriched genes (genes with higher relative expression in the striatum when compared to other brain regions) are decreased in a HD mouse model and in the caudate of HD patients (21). Down-regulation of novel striatal-enriched genes involved in vesicle transport and trafficking, tryptophan metabolism and neuroinflammation have also been identified in both HD mouse striatum and caudate from HD patients (22). Transcriptional dysregulation occurs in large genomic regions, in a coordinated fashion and is associated with disease progression. Hence genome-wide expression profiling of the blood from HD patients revealed significant differences in symptomatic patients (23) but not moderate-stage patients (20). Thus, these biomarkers need to be further validated before their widespread use in clinical trials.

## Pathogenic Interaction of Exp-HTT with Nuclear Proteins

Huntingtin has multiple interacting partners, among which are transcription factors or co-activators of the transcriptional machinery, some of them exhibiting enhanced binding with Exp-Htt, while a handful prefers binding with wild-type Htt (24, 25). Due to its polyglutamine expansion, Exp-Htt abnormally interacts with several proteins involved in transcription regulation. These include the global transcriptional regulator TATA-binding protein/TFIID (26), TAFII130, a co-activator involved in cAMP-responsive element binding protein (CREB)-dependent transcription (27). An abnormal interaction of Exp-Htt has also been shown with specificity protein 1 (Sp1) (28), p53, CREB binding protein (CBP) (29, 30), and nuclear receptor co-repressor (NCoR) (31). The global consequence of these pathogenic interactions is a widespread transcriptional dysregulation. Thus, overexpression of Sp1 and TAFII130 in cultured striatal cells reverses the transcriptional inhibition of the dopamine D2 receptor gene caused by Exp-Htt, and protects neurons from Exp-Htt-induced cellular toxicity (28). Exp-Htt induces upregulation of p53 and its downstream targets, Bax and Puma, both *in vitro* and in postmortem brains of HD patients (32, 33). This results in mitochondrial membrane depolarization and decreased complex IV activity. p53 inhibition or its genetic deletion ameliorates mitochondrial defects in HD cell cultures (33).

CRE-regulated genes have been well described for their role in neuronal survival (34) and impairment of CRE-dependent transcription can account for the neurodegenerative process in HD. One of the CRE-regulated genes that has been directly associated with striatal neuro-degeneration is the peroxisome proliferator-activated receptor co-activator-1α (PGC-1α), a transcriptional co-activator that controls the expression of genes involved in mitochondrial biogenesis, respiration and glucose/fatty acid metabolism (35). Exp-Htt is known to cause energy dysfunction that is mainly related to mitochondrial abnormalities (36–38). Expression of PGC1-α is down-regulated in HD patients and HD mice (39). This down-regulation is explained by an interference of Exp-Htt with the CREB/TAF4-dependent transcriptional pathway. Cross-breeding of *Pgc-1*α knock-out mice with HD knock-in mice leads to increased degeneration of striatal neurons and motor abnormalities in the HD mice, whereas lentiviral-mediated overexpression induces neuro-protection. Decreased expression of PGC1-α accounts for abnormal myelination in HD, since Exp-Htt-induced down-regulation of PGC1-α in oligodendrocytes leads to inhibition of genes involved in myelination (40). PGC1-α can also control extrasynaptic NMDAR activity in neurons, which contributes to excitotoxicity in HD (41). Suppression of PGC1-α contributes to Exp-Htt-induced increase in extrasynaptic NMDAR activity and vulnerability. Others key regulators of PGC-1α, are Mitogen and Stressed-activated protein Kinase-1 (MSK-1), and SIRT3. MSK-1 is a striatum-enriched nuclear protein kinase, targeted by the pro-survival Extracellular-signal Regulated Kinase (ERK) signaling pathway. By regulating CREB phosphorylation, along with histone H3 phosphorylation, MSK-1 is directly involved in the expression levels of PGC-1α, and as such protects against Exp-Htt-induced striatal death *in vitro* and *in vivo* (42, 43) (see below). SIRT3 is one the seven mammalian homologs of the sirtuin gene family. This mitochondrial deacetylase, initially described in brown adipocytes, regulates mitochondrial functions and thermogenesis (44). In response to exercise, SIRT3 controls CREB phosphorylation and PGC-1α expression, via AMP-activated protein kinase (AMPK) (45). Exp-Htt induces decreased deacetylase activity of SIRT3 and further leads to reduction in cellular NAD(+) levels and mitochondrial biogenesis in cells. Viniferin, a natural compound that activates AMPK and enhances mitochondrial biogenesis, is neuroprotective in HD cellular models, an effect that tightly depends on SIRT3 activity (46). Strikingly, the sirtuin family members seem to be intimately linked to pathogenesis in HD, since the NAD+-dependent deacetylase activity of SIRT1 is also involved in the regulation of transcription in HD. SIRT1 is a nuclear protein that normally controls CREB phosphorylation levels via TORC1 (Regulated transcription co-activator 1 (TORC1) activity (47, 48). By interacting with SIRT1, Exp-Htt inhibits its deacetylase activity, and causes hyperacetylation of TORC1. This results in a decrease of CREB-regulated genes, including BDNF, and probably PGC1-α.

Altogether, these data strongly support that transcriptional dysregulation in HD plays a major role in mitochondrial dysfunctions and energy metabolism deficit, two important hallmarks of the pathology.

## Impairment of Cytosolic Sequestration of REST

Wild-type Htt sequesters R element-1 silencing transcription factor (REST), a transcriptional repressor of neuronal survival factors, including brain-derived neurotrophic factor (BDNF). This neurotrophic factor is expressed by cortical neurons, which project to the striatum, and is critical for striatal survival. Interestingly, both transcriptional regulation and axonal transport of BDNF (49, 50) are altered in HD. Htt interacts with REST in the cytoplasm, and this interaction is impaired by Exp-Htt. Thus, increased nuclear translocation of REST is observed in the presence of Exp-Htt. Locally, REST exerts a potent inhibitory role on *Bdnf* transcription and other neuronal genes (49, 51, 52). In this context, the consequence of the loss of function of Htt is directly correlated with HD pathogenesis. Expression level of BDNF is decreased in the striatum of HD patients and in the cortex of HD mouse models (49, 53, 54). Down-regulation of BDNF in the striatum specifically worsens the HD phenotype, whereas elevating BDNF expression in the forebrain alleviates it (54–58). The role of REST in HD may not be restricted to the regulation of *Bdnf* transcription since several REST targets are known to be dysregulated in HD (52, 58). REST seems to have a widespread role on gene dysregulation in HD, since it also controls non-coding RNAs (see below). *In vivo* delivery of a dominant negative form of REST in the motor cortex restores the expression of BDNF mRNA and protein along with other REST-regulated genes in this region (59). Surprisingly, despite this important effect on gene regulation, no therapeutic effects were found in motor function in HD mouse models. These data raised the question as to whether a more widespread rescue of REST-regulated genes in the brain may be necessary.

## Chromatin Remodeling in Huntington’s Disease

Chromatin remodeling is an “above the genome” molecular mechanism, that gates DNA access, and hence transcription. It is critically controlled by post-translational modifications (PTM) of histones (H2A and H2B, H3 and H4), a group of highly basic proteins tightly linked to DNA. By modifying the electrostatic interactions between the N-terminal domain of histones and DNA, PTM of histones contribute to the chromatin structure, and access of the transcriptional machinery to the DNA (60). In particular, the methylation or acetylation state of histones is closely linked to regions of transcriptional activity, by regulating transcription factor access to promoter regions in the DNA.

The enzymes that catalyze histone acetylation are histone acetyltransferases (HATs) whereas Histone Deacetylases (HDACs), catalyze the reverse deacetylation reaction (60–62). By interacting with CBP and p300/CBP-associated factor (P/CAF), Exp-Htt blocks their intrinsic HAT activity (29, 30, 63, 64). This results in a global reduction of histones H3 and H4 acetylation levels, along with CBP-regulated gene transcription. Overexpression of CBP reduces Exp-Htt-induced toxicity (30).

Determining experiments were performed to demonstrate that HDAC inhibitors (HDACis), including SAHA, sodium butyrate, or phenylbutyrate improved behavioral performance and increased neuronal survival in several HD models (64–67). These data lead to the general concept that HDACi could be a new therapeutic avenue in HD. This concept is however weakened by the toxicity of the aforementioned HDACi compounds at therapeutic doses. Furthermore, it must be emphasized that the levels of acetylated histones are not decreased globally in HD mouse models, but rather selectively in the promoters of genes that are specifically down-regulated in HD (68).

So far, HDACis act broadly on the HDAC family, which comprises 11 members divided into four classes: I (HDAC1, 2, 3 and 8), IIa (HDAC4, 5, 7 and 9), IIb (HDAC6 and 10), and IV (HDAC11) (69). Their relative toxicity can be due to either inhibition of a pro-survival HDAC isoform, or low substrate specificity, a single enzyme being capable of deacetylating multiple sites within histones (60). Thus, it was postulated that inhibitors targeting one specific HDAC might produce a better benefit to side effect ratio.

To unravel this issue, genetic invalidation of each single HDAC was investigated in the R6/2 mouse model. These studies revealed that reduction of Hdac3, 5, 6, 7, and 9 expression had no effect on HD-related phenotype (70–72), whereas reduction of Hdac4 expression showed a significant beneficial effect (73). This raises the interesting question as to whether specific HDAC4 inhibitors may be more adapted for HD treatment, an issue that is now under investigation (73).

Methylation of histones affects lysine and arginine residues and is associated to either activation or repression of transcription, depending on the modified residues. One of the proteins involved in methyltransferase activity at histone H3 (K9) is ERG-associated protein with SET domain (ESET). ESET expression is increased in HD patients and R6/2 HD mice (74). Sp1 acts as a transcriptional activator of the ESET promoter at guanosine-cytosine (GC)-rich DNA binding sites (75). Inhibiting Sp1 binding to these sites using mitramycin (a clinically approved antitumor antibiotic) suppressed basal ESET promoter activity in a dose dependent manner and lead to extended survival, enhanced motor performance and improved brain histopathology in R6/2 mice (74). Interestingly, the reduction of H3K9 hypermethylation induced by mithramycin or chromomycin, is associated with an increased acetylation of the same residue (76). On the other hand, the beneficial effect of the HDACi phenylbutyrate in HD mice is accompanied by an increase in H3 and H4 acetylation and a concomitant decrease in H3 methylation (67). These data illustrate that a crosstalk between acetylation and methylation participates to the nucleosomal dynamics, and that disequilibrium between these two epigenetic marks can be corrected by the inhibition of either deacetylation or methylation.

Monoubiquitylation of histones has also been implicated in HD-related transcriptional dysregulation. This modification, which involves E3-ubiquitin ligase complexes, affects lysine residues of histone H2A (uH2A) and H2B (uH2B) and is associated to either activation or repression of transcription depending on the modified residues. Exp-Htt expression alters the activity of specific E3-ubiquitin ligases, and modifies uH2A and uH2B. Knocking down the H2A E3-ubiquitin ligase reduces uH2A and rescues transcriptional repression in Exp-Htt knock-in cells. In contrast, knocking down the H2B E3-ubiquitin ligase induces transcriptional repression in wild-type Htt knock-in cells (77).

Core histones can be post-translationally modified by transglutaminases (TG), which catalyze transamidation of glutamine residues. All four mammalian core histones, H2A, H2B, H3, and H4, were shown to be glutaminyl substrates of TG2, a nuclear TG, and their crosslinking contributes to chromatin condensation *in vitro* (78–80). Total TGs activity is elevated in brain extracts from HD patients (81) and treatment of R6/2 mice with a TG competitive inhibitor, cystamine, extends survival, reduces tremor and abnormal movements and ameliorates weight loss in these mice (82). Therefore, TGs were suggested to participate to chromatin remodeling and gene expression dysregulation in HD. McConoughey and colleagues showed that TG2 polyaminates H3 N-terminal tail which increases its positive charge and therefore its propensity to more tightly interacts with DNA (83). TG2 occupies the promoter/enhancer regions of two genes essential for energy production, PGC1-α and cytochrome *c*, and a selective inhibition of TG2 in a HD striatal cell line corrects gene dysregulation. Therefore TG2 inhibition has emerged as a HDAC-independent epigenetic therapeutic strategy for HD.

**Figure 1:**
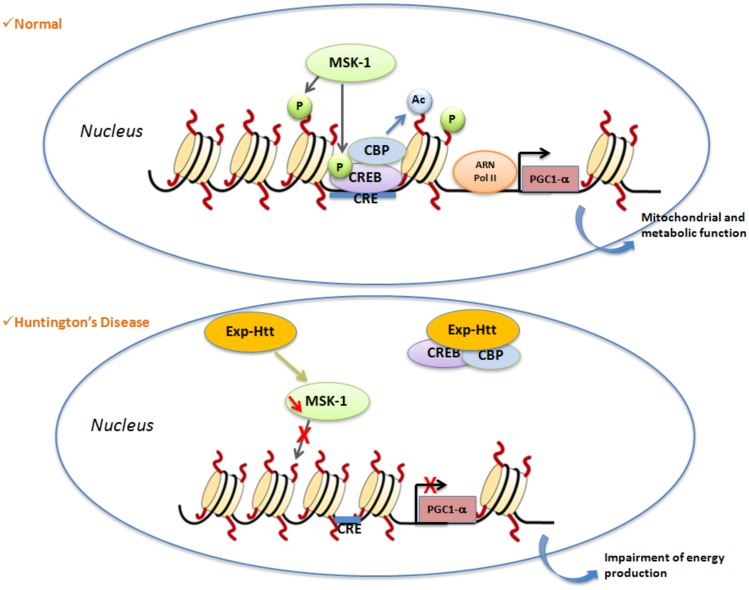
**Illustration of a signaling pathway involved in histone PTMs in HD**. Under normal conditions, activation of nuclear MSK-1 induces phosphorylation of (1) histone H3 and (2) the transcriptional factor CREB. Phosphorylated CREB recruits CBP, and activates its histone acetyl transferase (HAT) properties. Post-translational modifications (PTM) of histones (phosphorylation, acetylation) lead to modification of chromatin structure from heterochromatin to euchromatin, which allows gene transcription to occur. One of the key genes regulated by CREB is *PGC1α*, a crucial gene involved for the regulation of mitochondrial and metabolic functions. In Huntington’s disease, Exp-Htt expression results on (1) down-regulation of MSK-1 expression and (2) sequestration of CREB and CBP within Exp-Htt aggregates. Therefore, the heterochromatin structure is favored, a structure that impairs transcription of CREB target genes.

Histone phosphorylation is mainly described as an activating chromatin mark of gene activation. This PTM affects serine, threonine and tyrosine residues. Histone phosphorylation is controlled by the interplay between kinases and phosphatases that respectively add and remove phosphate onto each of these residues. Histone kinases phosphorylate the hydroxyl group of the targeted amino-acid side chain therefore leading to a change of the global charge of histones, a reduced interaction between histones and DNA and a relaxation of chromatin (60). Phosphorylation of H3S10 involves MSK-1 that was shown to be down-regulated in HD cells, mice and patients (42). Restoration of MSK-1 expression in striatal neurons *in vitro* and in the lentiviral-based rat model of HD protects against neuronal dysfunctions induced by Exp-HTT (42, 43). In contrast, MSK-1 knock-out mice exhibit spontaneous striatal atrophy when they age, and a higher sensitivity to the 3-nitropropionic acid (3NP), a mitochondrial neurotoxin that induces selective degeneration of striatal neurons and HD-like symptoms in humans, monkeys, and rodents (43). In addition to its H3S10-kinase activity, MSK-1 phosphorylates, and activates CREB, leading to the regulation of PGC-1 α, both *in vitro* and *in vivo* (Figure 1).

In addition to histone PTMs, chromatin remodeling is controlled by DNA methylation. A recent study showed that Exp-HTT induces an extensive alteration of DNA methylation on a large proportion of genes that change in expression in HD (84). The authors of this study identified more specifically two transcriptional regulators, AP-1 and Sox2, associated with DNA methylation changes. Since these epigenetic changes are more stable, this could explain the long-term modifications of gene expression in HD.

## HD and miRNAs

About 98% of human transcribed genome is dedicated to non-protein-coding RNAs (NcRNAs) genes, with regulatory properties on gene expression. Among ncRNAs, microRNAs (miRNAs) are 21–23 nucleotide RNA molecules that regulate gene expression by promoting either degradation or translational-inhibition of target mRNAs (85). The miRNA pathway starts in the nucleus with the RNA polymerase II-mediated transcription of primary (pri-miRNAs) hairpins, which are cleaved into precursor of miRNAs (pre-miRNAs) by the nuclear proteins Drosha and DiGeorge syndrome critical region 8 (DGRC8) (86, 87). Pri-miRNAs are then transported to the cytoplasm (88, 89) and processed into 22nt duplex mature miRNAs by the RNAseIII Dicer (90), which is then assembled into the RNA-induced silencing complex (RISC) with the protein Argonaute (Ago) (91). MiRNAs suppress post-transcriptional expression of genes by guiding RISC interaction with their specific sequence motifs within the 3′untranslated region (3′UTR). This results in either degradation or translational-inhibition of the target mRNAs (92–95).

Many miRNAs are selectively and abundantly expressed in the CNS where they play key roles in the elaboration of the neuronal transcriptome (96) and seem to be important mediators of plasticity (97). MiRNA dysregulation has been associated with several human disorders of the CNS. The first evidence came from studies showing that Dicer or DGCR8 ablation impairs neuronal differentiation, produces synaptic dysfunctions, disturbs axonal path-findings, and induces neuro-degeneration, suggesting that miRNAs play important roles in neurological disorders (98, 99). Evidence of miRNA dysregulation in HD exists. Two different and complementary aspects of this dysregulation arise from the recent literature. First, there is now increasing evidence that multiple neural miRNAs are decreased in HD neurons, with concomitant de-repression of downstream target mRNAs (58, 100–102). Second, several elegant studies demonstrate that Exp-HTT protein (103) or RNA (104) may play a major role in the processing of miRNAs and hence pathogenesis.

**Figure 2:**
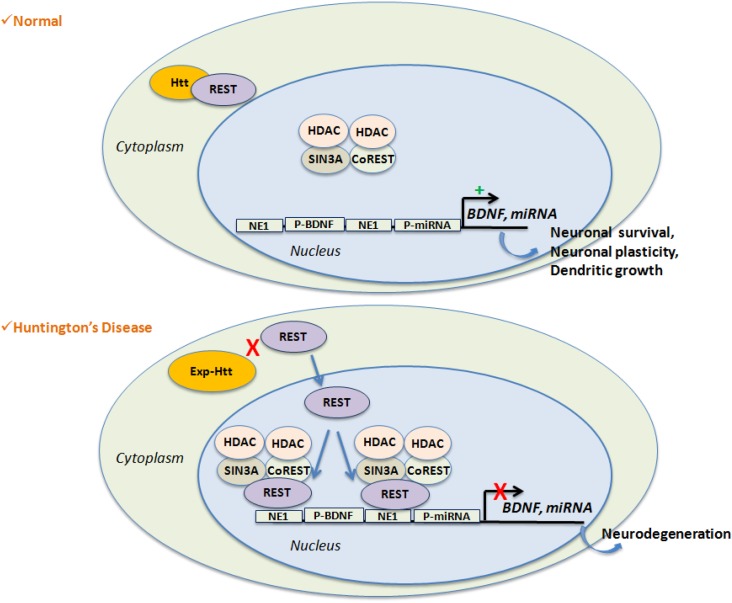
**REST-mediated gene repression is favored in HD**. Wild-Type Htt interacts with REST, a transcriptional repressor, within the cytoplasm. This leads to de-repression of REST target genes like *BDNF* or non-coding miRNAs, which are essential for neuronal survival, neuronal plasticity or dendrites growth. Expansion of Htt disrupts its binding with REST and facilitates nuclear entry of REST along with the formation of the repressor complex on the RE1 site. Activation of RE1 site results on target gene silencing and participates to neuro-degeneration.

Using an *in silico* approach, the group of Cataneo identified 17 miRNA genes as likely targets of REST (58) (Figure 2). The regulation of these miRNAs by REST was evaluated in embryonic striatal cell lines, and mir-29a, mir-124a, mir-132, and mir-135b were shown to be significantly upregulated upon loss of REST function and in the cortex of 12-week-old R6/2 mice. In humans, mir-132 expression level is significantly lower in HD samples compared to control. In contrast, mir-29a and mir-330 expression is significantly higher in HD samples. Packer et al. (102) used a screen of predicted REST-regulated miRNAs from HD patient brain samples, and found significant decreases of miR-9, miR-9*, and miR-29b as well as a significant increase of miR-132 at late stages. They also found that the bi-functional brain enriched miR-9/miR-9* targets two components of the REST complex: miR-9 targets REST and miR-9* targets CoREST. A characterization of miRNAs profiling and sequence modification was performed by Illumina sequencing in the frontal cortex and the striatum. It showed a strong deregulation of miRNA and IsomiRs (miRNAs containing length and sequence heterogeneity) in HD, most being common to both frontal cortex and striatum (105). Of interest, the co-regulated miRNAs contained regulatory sequences for REST and p53, suggesting a key role of these genes in down-regulation of gene expression in HD. Profiling of miRNAs expression was also performed in the YAC128 and R6/2 mice, showing that nine miRNAs (miR-22, miR-29c, miR-128, miR-132, miR-138, miR-218, miR-222, miR-344, and miR-674*) are commonly down-regulated in 12-month-old YAC128 mice and 10-week-old R6/2 mice (100). Concomitantly, the expression of Dicer is decreased at the late stages in these two mouse lines, indicating that miRNA biogenesis is altered in HD. More recently, Soldati and collegues, found the same results in HD cell lines (101). Rescuing miR-22 expression in *in vitro* HD models, protected against Exp-Htt-induced neurotoxicity (106). Very recently, miR-196a was shown to reduce the expression of Exp-HTT *in vitro*, and to improve molecular, pathological, and behavioral phenotypes in a HD transgenic mouse model (107). Of importance, miR-196a ameliorated the formation of aggregates in iPSC (inducible Pluripotent Stem Cells) from HD patients, when differentiated in the neuronal stage. The down-regulation of Exp-HTT by miR-196a is of prime importance, since it forms the bases of new strategies for allele-specific silencing in HD. miR-196a did not regulate Exp-Htt levels directly, but rather indirectly, probably through the regulation of the ubiquitin-proteasome system, gliosis, and the CREB pathway.

Additionally, HTT has a more direct role in post-transcriptional gene silencing by miRNAs. An elegant study from Naoko Tanese’s group (103) has shown that HTT itself contributes to RNA-mediated gene silencing through its association with Ago in Processing bodies (P-bodies). Mouse striatal cells expressing Exp-Htt showed fewer P-bodies and reduced reporter gene silencing activity compared to wild-type. More recently, a pathogenic role of the Exp-*HTT* RNA was provided (104). The authors showed that Exp-*HTT* mRNA generates small CAG-repeated RNAs (sCAGs) having a neurotoxic activity. This toxic effect was dependent on Dicer and Ago proteins, as they were inhibited by their knock-down. They thus provide the first demonstration that these sCAGs generated by Exp-HTT may contribute significantly to the neuro-degeneration pattern observed in HD.

## Conclusion

Most of the cellular dysfunctions in HD are due to alterations of gene expression: from mitochondrial dysfunctions and metabolism energy deficit, to excitotoxicity. Furthermore, dysregulation of transcription is a widespread, reproducible, and early event in the pathogenic process of HD. Therefore, new therapeutic approaches targeting transcription factors, chromatin remodeling, or miRNAs can be proposed. Obviously targeting signaling pathways that control expression levels of the trophic factor BDNF or the mitochondrial gene PGC1-α will provide interesting perspective. Targeting the REST transcriptional repressor, CREB, or Sirtuins remain interesting strategies. Although therapeutic trials, including safety and tolerability studies, with the global HDACi, phenylbutyrate, have been conducted in patients, these compounds remain highly unspecific, since they act on multiple classes of HDACs, hence on numerous non-selected genes and sometimes non-nuclear targets. Alternative approaches could be to design compounds that target more specifically one type of HDAC, for example HDAC4 – an issue that is under investigation – or to target other PTMs of histones (including phosphorylation, methylation, ubiquitylation, or polyamination), each PTM targeted alone, or in combination. It was recently discovered that non-coding RNAs are dysregulated in HD. Because one miRNA can target multiple pathways, this suggests that miRNAs could have pleiotropic, widespread effects on HD pathogenesis. An elegant demonstration of this assumption was recently made both *in vitro* and *in vivo*, using miR-196a, including in IPSC from HD patients. One important finding in this regard was that Exp-HTT itself was down-regulated by miR-196a. Therefore, a new and fascinating therapeutic avenue is now offered with miRNAs in HD.

## Conflict of Interest Statement

The authors declare that the research was conducted in the absence of any commercial or financial relationships that could be construed as a potential conflict of interest.
